# Genome-Wide Identification of the Soybean GH5 Gene Family and Functional Analysis of *GmGH5-22* in Salt Tolerance

**DOI:** 10.3390/plants15172700

**Published:** 2026-09-02

**Authors:** Xi Chen, Lingshan Ren, Naize Mu, Xiaoxuan Guo, Wanhong Li, Bowei Jia, Jianwei Li, Yan Wang, Yang Shen, Xiaoli Sun, Mingzhe Sun

**Affiliations:** 1 Crop Stress Molecular Biology Laboratory, College of Agriculture, Heilongjiang Bayi Agricultural University, Daqing 163319, China; chenxi521415@163.com (X.C.); sansan891213@163.com (L.R.); 13563346705@163.com (N.M.); guoxiaoxuan0619@163.com (X.G.); wh10161122@163.com (W.L.); jiabowei_paper@163.com (B.J.); ljw_plant@yeah.net (J.L.); 18645232886@163.com (Y.W.); 15603692106@163.com (Y.S.); sunxiaoli2016@byau.edu.cn (X.S.); 2 Beidahuang Kenfeng Seed Co., Ltd., Harbin 150030, China; 3 College of Agriculture, Northeast Agricultural University, Harbin 150030, China

**Keywords:** *Glycine max*, GH5 family, stress response, salt tolerance

## Abstract

Plant GH5 family genes function in both cell wall biosynthesis and stress responses. However, comprehensive studies on GH5 genes in the soybean remain limited. Here, we identified 28 *GmGH5* genes from the soybean genome. Phylogenetic analysis assigned these genes to three subfamilies (I–III), with no representatives in subfamily IV. The GmGH5 family harbors 15 conserved motifs, which are largely similar within subfamilies but differ across subfamilies. Additionally, exon–intron structures (2–7 introns) exhibit clade-specific patterns, with members within the same clade sharing similar intron numbers and lengths, whereas distinct clades show some variation. The promoter regions of *GmGH5* genes contained various cis-acting regulatory elements associated with stress responses and developmental processes. Transcriptome-based expression profiling revealed distinct tissue-specific expression patterns of *GmGH5* genes. RT-qPCR further confirmed their differential expression under salt, alkaline, cold, and drought stresses, especially a significant increase in *GmGH5-22* expression under salt stress (approximately 22-fold at 6 h, **** *p* < 0.0001). Furthermore, *GmGH5-22* was highly expressed in roots, and transient expression in tobacco leaves showed its peripheral localization, which aligns with its predicted extracellular localization, suggesting that GmGH5-22 is highly likely localized to the cell wall. Overexpression of *GmGH5-22* in soybean hairy roots significantly improved tolerance to salt stress. These findings establish a foundation for functional characterization of *GmGH5* genes and provide viable targets for molecular breeding to enhance salt tolerance in soybeans.

## 1. Introduction

Soil salinization represents a primary abiotic stress factor that undermines global agricultural sustainability and food security. According to the global assessment report released by the Food and Agriculture Organization (FAO) in 2024 [1], approximately 1.38 billion hectares, accounting for nearly 10.7% of the total land area worldwide, are currently affected by salinization, with an additional one billion hectares at risk from the climate crisis and inappropriate anthropogenic management. Salinization has already compromised approximately 20% of irrigated croplands globally, and in severely impacted areas, yield reductions for sensitive crops such as rice and legumes can reach up to 70%.

The soybean (*Glycine max* (L.) Merr) is an important source of vegetable oil and protein worldwide [2]. However, its productivity and cultivation are severely constrained by soil salinization [3]. Salt stress triggers a cascade of interconnected physiological disturbances in soybeans, including osmotic imbalance, ionic toxicity from excess Na^+^ accumulation, and oxidative damage from reactive oxygen species (ROS) bursts [4,5]. Together, these impairments inhibit both vegetative growth and reproductive development, culminating in substantial yield reductions under saline conditions [6,7]. Salt tolerance in soybeans operates through ion homeostasis, osmotic adjustment, and ROS scavenging, executed by ion transporters, compatible solutes, and antioxidant enzymes [3,4,5].

Beyond ion homeostasis, osmotic adjustment, and ROS scavenging, the plant cell wall has emerged as a key determinant of stress adaptation [8,9]. Serving as the first barrier sensing environmental cues, the cell wall undergoes dynamic remodeling of its composition and architecture to maintain cellular integrity under adverse conditions [10,11,12,13]. Glycoside hydrolases (GHs) have emerged as pivotal regulators that orchestrate this dynamic remodeling, a process fundamental to growth, development, and stress responses [14,15,16,17]. Among the various GH families, Glycoside hydrolase family 5 (GH5) is one of the most functionally diverse [18]. GH5 comprises enzymes that share a conserved (β/α)_8_ barrel catalytic domain, yet they display considerable functional diversity, including β-mannanase, β-glucanase, and β-glucosidase activities [18,19]. GH5 enzymes hydrolyze glycosidic bonds in cell wall polysaccharides, releasing oligosaccharide fragments that are perceived as damage-associated molecular patterns (DAMPs) to activate stress signaling [20,21]. Under salt stress, this hydrolysis promotes cell wall remodeling by loosening wall architecture to sustain root growth, while simultaneously releasing soluble sugars that contribute to osmotic adjustment and bolster antioxidant defense [22]. Thus, GH5 enzymes bridge physical cell wall modification with systemic stress responses, positioning them as key players in salt tolerance. Although the family is phylogenetically subdivided into multiple lineages, many GH5 members have been functionally linked to plant cell wall metabolism and stress responses. In rice, a stress-inducible GH5 β-glucosidase carrying a fascin-like domain was identified, suggesting a possible role in carbohydrate binding or sensory functions [23]. Other rice GH5 genes have been associated with seed development and endosperm traits [24]. Genome-wide surveys in foxtail millet and grape have further documented the presence and differential expression of GH5 members, indicating their evolutionary conservation across monocots and dicots [25,26]. At the structural level, a recently characterized soybean GH5 β-mannanase has provided detailed insights into substrate recognition and acidophilic catalysis [27]. Together, these findings highlight GH5 enzymes as versatile players in cell wall remodeling, reproductive development, and stress responses.

Despite the recognized importance of GH5 family members in plant growth and abiotic stress responses, their functional roles in the soybean remain largely elusive, particularly under salt stress. In this study, genome-wide identification of the soybean *GmGH5* gene family revealed *GmGH5-22* as a positive regulator of salt stress response. *GmGH5* members are evolutionarily conserved with tissue-specific and stress-inducible expression. Overexpression of *GmGH5-22* in soybean hairy roots enhanced salt tolerance. These findings provide a basis for elucidating the regulatory mechanism of *GmGH5-22* in stress responses and highlight its potential as a candidate gene for improving stress tolerance in the soybean.

## 2. Results

### 2.1. Identification of the GmGH5 Family in the Soybean

Through systematic genome-wide analysis using HMMER3.0, a total of 28 non-redundant GH5 genes were identified in the soybean genome. These genes were designated as GmGH5-1 through GmGH5-28 according to their chromosomal order. The complete coding sequences (CDSs) of the *GmGH5* genes span 1209 to 1728 base pairs. The physicochemical characteristics of the 28 GmGH5 proteins were then systematically characterized. Table 1 summarizes the essential features of the GmGH5 family. The lengths of sequences range from 402 (GmGH5-15) to 575 (GmGH5-12) amino acids, and their molecular weights extend from 46.058 (GmGH5-15) to 63.148 (GmGH5-5) kDa, indicating considerable structural differences, potentially associated with genome size and species evolution. The isoelectric point falls within the range of 5.24 (GmGH5-13) to 9.54 (GmGH5-16). Among these, 20 of the 28 GmGH5 proteins (71.4%) had a pI below 7, suggesting that most GmGH5 members are rich in acidic amino acids. Protein architecture analysis revealed that 23 GmGH5 members featured an N-terminal signal peptide, whereas five members (GmGH5-6/9/19/20/23) lacked this feature but contained transmembrane domains (9–35 aa). GmGH5-12 and GmGH5-17 additionally carried an RICIN (R-type lectin) domain, pointing to a modular secretory glycoside hydrolase organization potentially associated with cell wall polysaccharide binding.

Subcellular localization prediction revealed diverse compartmental distributions among the 28 GmGH5 members (Table 1). Among these, 16 showed cytoplasmic localization (14 exclusively and 2 with additional localizations), whereas 10 were localized to the cell wall (6 exclusively and 4 with additional localizations), and the remaining 2 were solely nuclear-localized.

### 2.2. Chromosomal Location and Phylogenetic Analysis of the GmGH5 Genes in the Soybean

**Figure 1:**
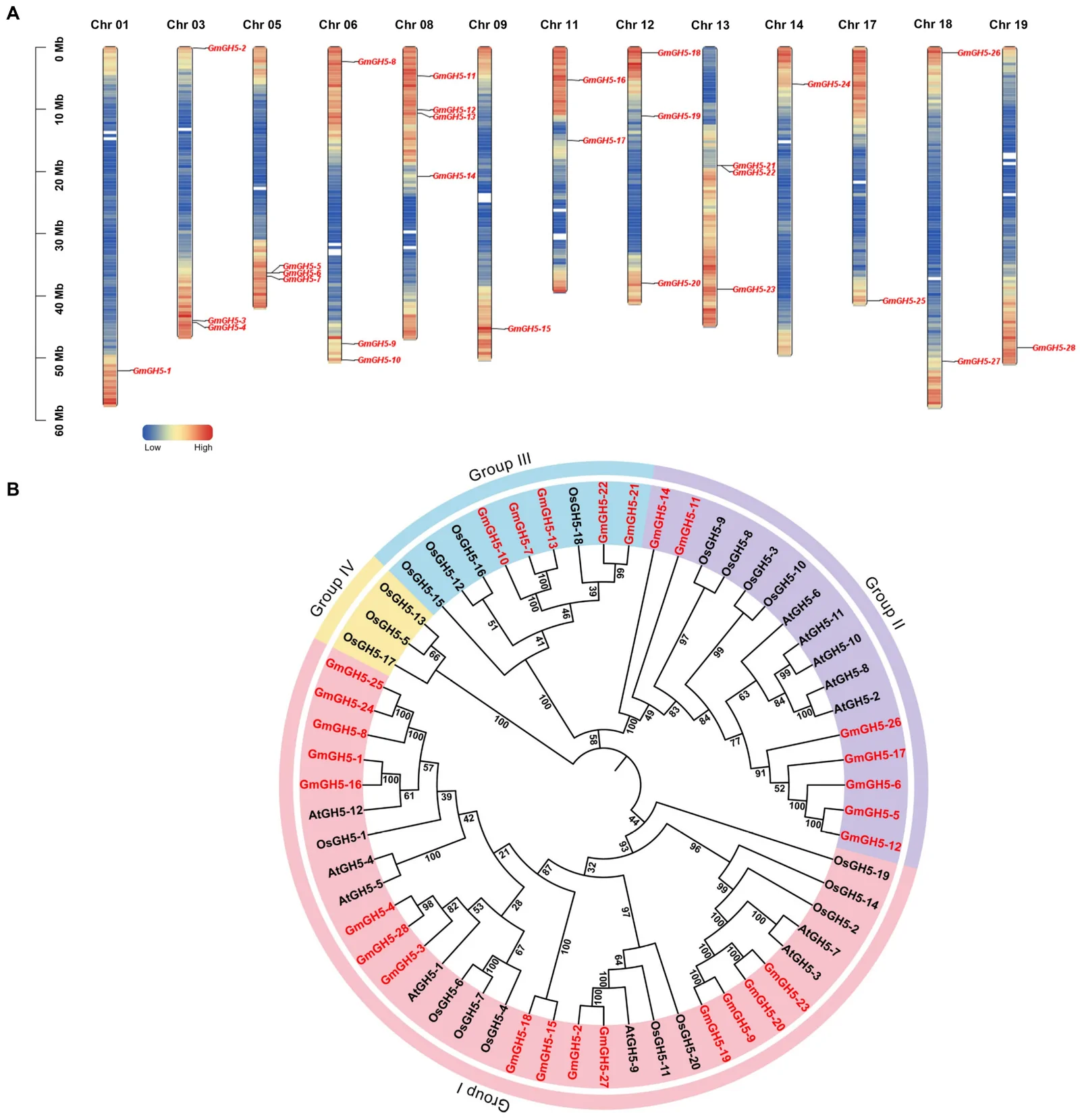
Chromosomal localization and phylogenetic analysis of GmGH5 genes. (**A**) Chromosome localization of 28 GmGH5 genes in the soybean (*Glycine max*) genome. The left vertical scale indicates chromosome lengths in megabases (Mb), and chromosome numbers are shown above each chromosome bar. (**B**) Phylogenetic tree of GmGH5 proteins from *Arabidopsis thaliana* (12), *Oryza sativa* (20), and *Glycine max* (28), constructed using the neighbor-joining (NJ) method based on full-length protein sequences. Red font indicates soybean GH5 gene family members.

These genes were distributed across 13 of the 20 soybean chromosomes (Chr 01, 03, 05, 06, 08, 09, 11, 12, 13, 14, 17, 18, and 19) (Figure 1A). Chromosome 8 harbored the highest number of GmGH5 genes (4 members). Chromosomes 03, 05, 06, 12, and 13 each contained 3 members, while the remaining chromosomes carried 1–2 members. Most GmGH5 genes were located near the distal ends of chromosomes, with only a few positioned in the central regions. Several GmGH5 members were arranged in tandem or tight clusters on the same chromosome (e.g., GmGH5-3 and GmGH5-4 on Chr03; GmGH5-5, GmGH5-6, and GmGH5-7 on Chr05).

Phylogenetic analysis of 60 GH5 proteins from three species of different affinities was performed, including 28 from *Glycine max*, 12 from *Arabidopsis thaliana*, and 20 from *Oryza sativa* (Figure 1B). The results of the phylogenetic analysis indicated that these GH5 proteins were classified into four distinct subgroups (Group I–IV). The 28 soybean GH5 genes were distributed unevenly among Groups I–III, with no soybean member in Group IV. Specifically, Group I contained the largest number of *GmGH5* genes (16 members, accounting for 57.1% of all GmGH5s), followed by Group II (7 members) and Group III (5 members). In Arabidopsis thaliana, only Group I and Group II members were identified, with no homologs in Group III or Group IV. These findings indicate that the four-group topology predates the monocot–dicot divergence, with Group IV retained in monocots but lost in dicots, and Group III also absent in Arabidopsis. The expansion of Group I in the soybean (16/28) further suggests a dicot-specific functional divergence of this subgroup.

### 2.3. Synteny Analysis of the GmGH5 Gene Family

**Figure 2:**
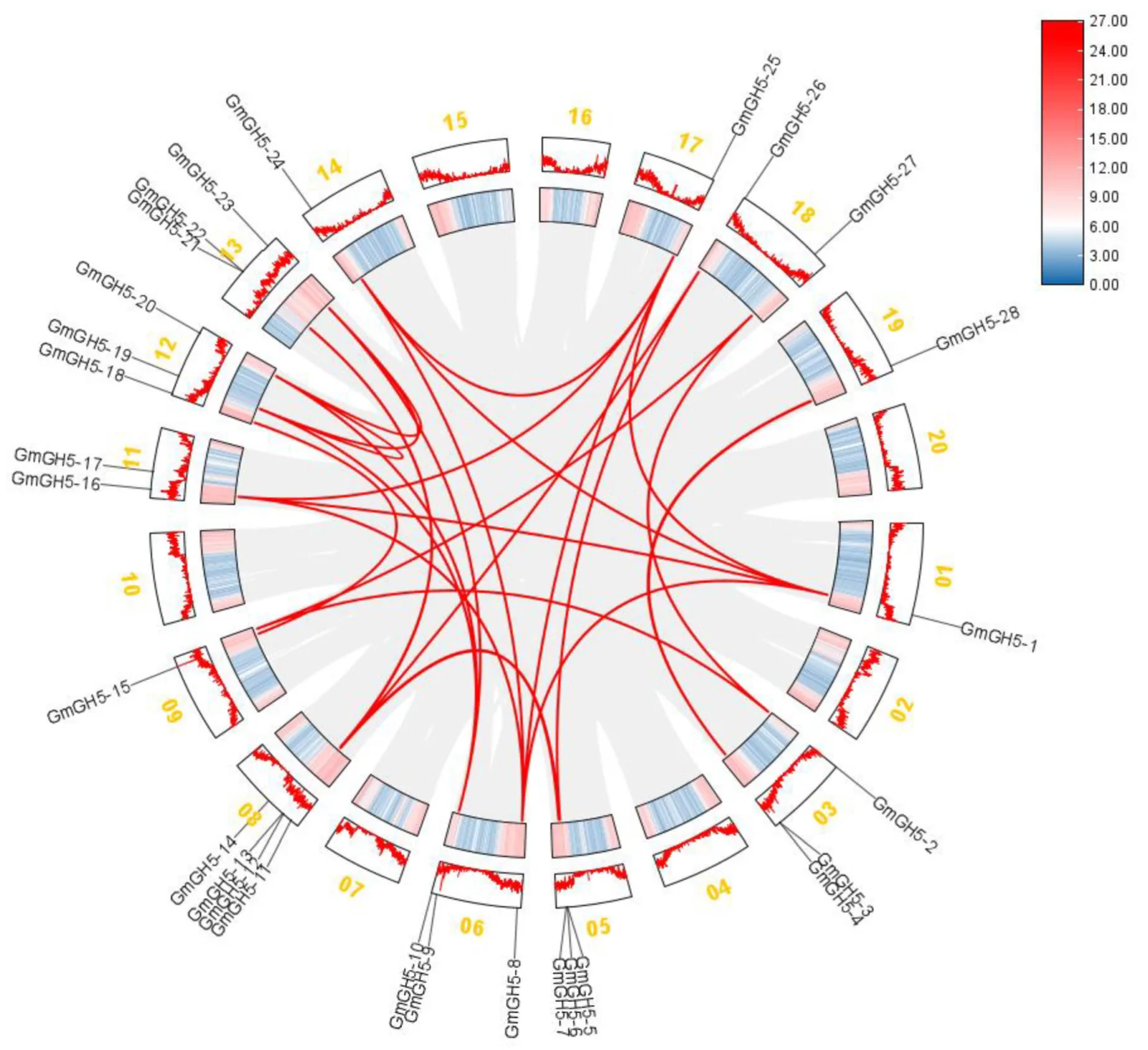
Collinearity analysis of GH5 genes in the soybean genome. The outermost ring (with red markers) indicates the physical loci of the *GmGH5* genes. The inner concentric track displays the gene density along each chromosome, represented by a line chart and a color scale ranging from blue (0.00) to red (27.00). In the center of the plot, gray lines represent collinear blocks between chromosomes, while red lines indicate segmentally duplicated gene pairs.

To investigate the evolutionary mechanisms underlying the expansion of the *GmGH5* family, we performed a synteny analysis and identified 24 segmental duplication gene pairs across the soybean genome. These pairs were distributed on 13 chromosomes, suggesting that segmental duplications have played a substantial role in the expansion of this family (Figure 2). For instance, robust duplication pairs were detected between *GmGH5-1* (Chr 01) and *GmGH5-16* (Chr 11), as well as between *GmGH5-2* (Chr 03) and *GmGH5-27* (Chr 18). Additionally, interconnected gene modules were also identified, such as a cluster linking *GmGH5-1* (Chr 01), *GmGH5-8* (Chr 06), *GmGH5-16* (Chr 11), *GmGH5-24* (Chr 14), and *GmGH5-25* (Chr 17), further supporting the retention of duplicated genes following whole-genome duplication (WGD) events. Taken together, these results indicate that inter-chromosomal segmental duplications, rather than intra-chromosomal events, served as the primary driving force for the expansion of the *GmGH5* family.

### 2.4. Analysis of Gene Structure and Protein Motif

**Figure 3:**
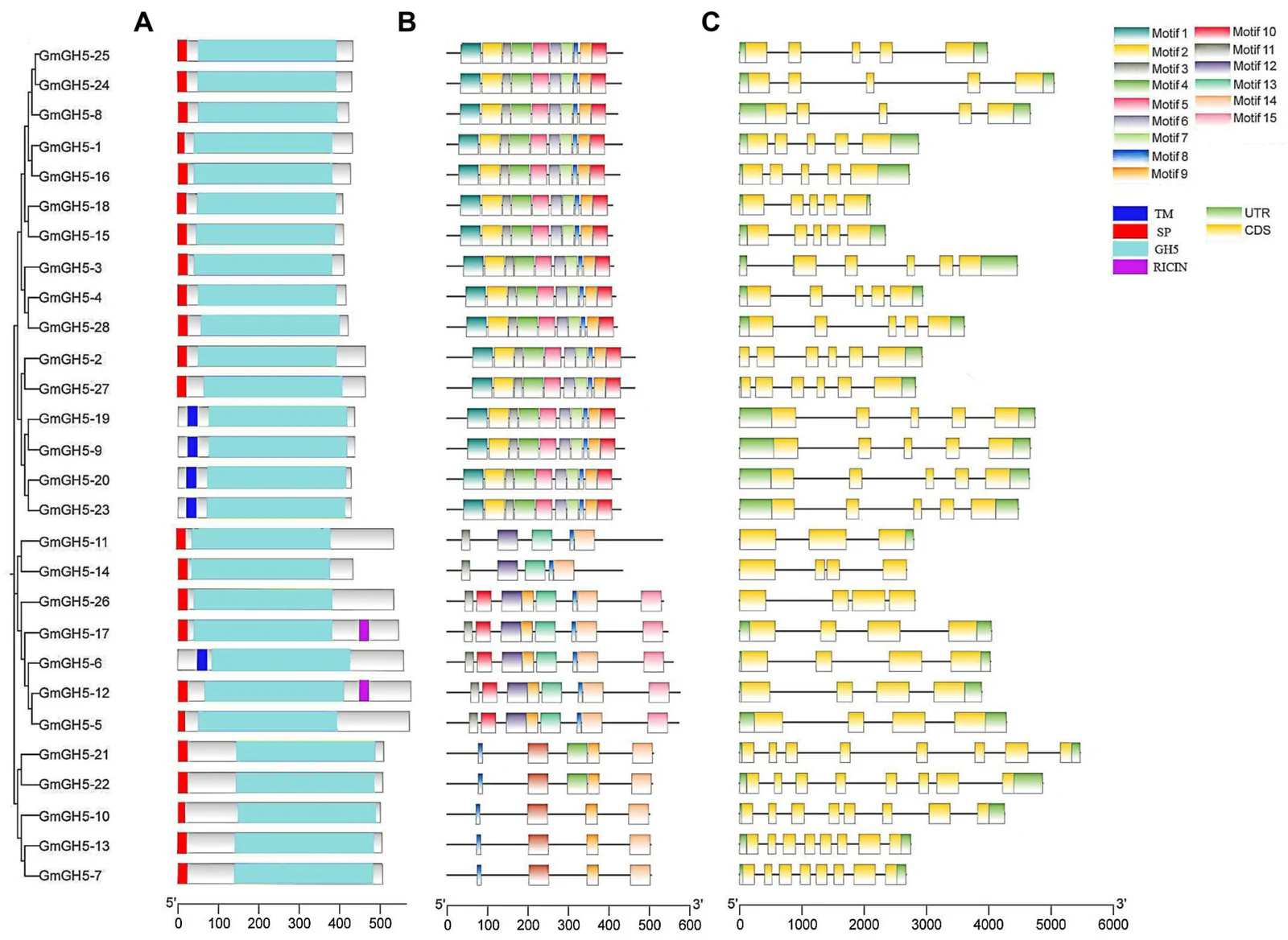
Conserved domains, gene structures, and conserved motifs of GmGH5s. (**A**) Conserved domain architectures. (**B**) Conserved motifs of GmGH5s, with black lines denoting the length of the protein sequences. (**C**) The genetic structure of *GmGH5s*. The UTR (untranslated region), CDS (coding sequence or exons), and introns are symbolized by green rectangles, yellow rectangles, and black lines, respectively.

To investigate the evolutionary diversity of the GmGH5 gene family, we analyzed conserved motifs using the MEME website and constructed an integrated diagram of conserved domains, gene structures, and motif distributions for the 28 GmGH5 proteins using TBtools-II, Version 128 (Windows-x64) (Figure 3). All members contained a complete GH5 catalytic domain. Most members contained an N-terminal signal peptide, whereas GmGH5-19, GmGH5-9, GmGH5-20, GmGH5-23, and GmGH5-6 harbored transmembrane domains instead. GmGH5-12, and GmGH5-17 contained an additional C-terminal RICIN domain (Figure 3A). MEME analysis predicted 15 conserved motifs (Motif 1–15). Members within the same clade shared similar motif compositions, whereas distinct subclades exhibited clear motif variations, suggesting that members of the same subclade may perform similar functions (Figure 3B). The *GmGH5* genes contained 2 to 7 introns, with notable variation in both intron number and length. However, this variation was largely subfamily-dependent, genes within the same subfamily shared similar intron structures, whereas those from different subfamilies exhibited marked differences (Figure 3C). Taken together, the observed variations in gene structure, motif composition, and domain distribution suggest functional divergence during the evolution of the *GmGH5* family, implying that different subgroups may assume distinct biological roles in planta.

### 2.5. Analysis of Cis-Acting Components

**Figure 4:**
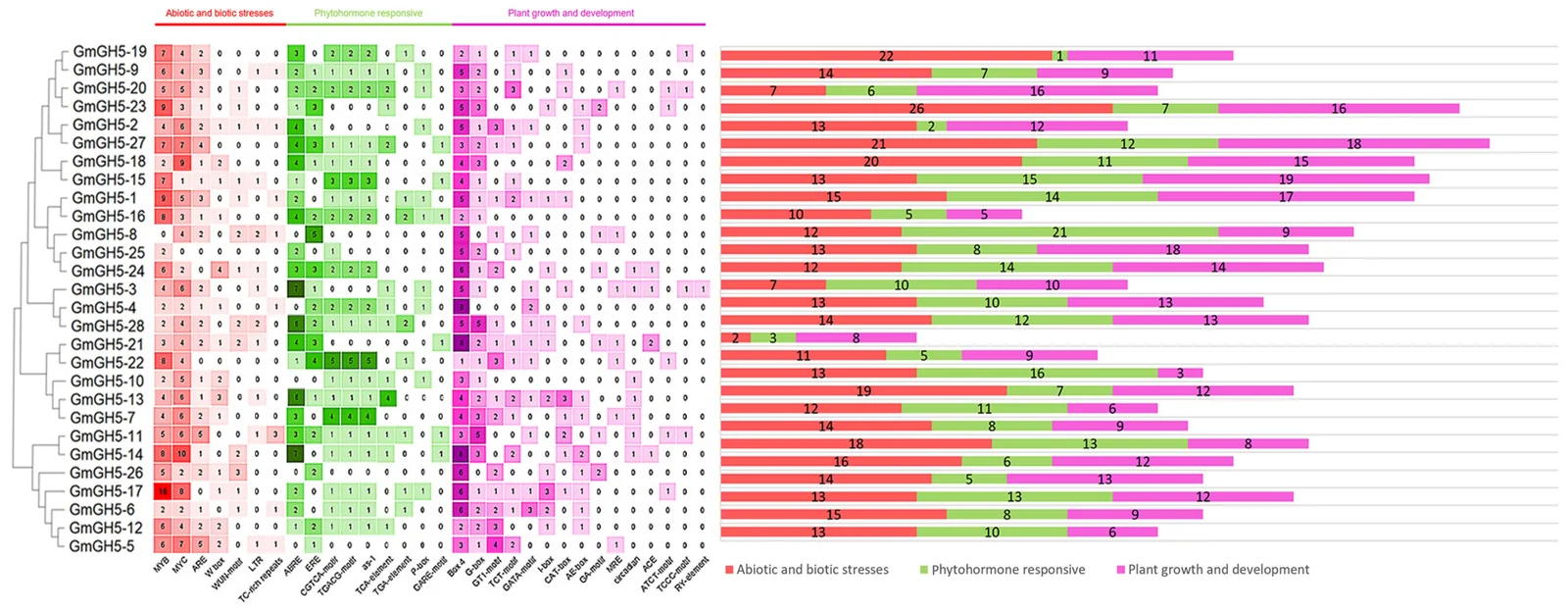
Predicted cis-elements in the promoters of GmGH5 genes. The types and numbers of cis-elements are indicated.

Promoter analysis of 28 *GmGH5* genes identified three major categories of cis-acting elements: stress-responsive elements (377 copies) were the most abundant, followed by plant growth-related elements (301 copies) and phytohormone-responsive elements (246 copies) (Figure 4). Further analysis revealed that among the stress-responsive elements, MYB, MYC, and anaerobic response elements (AREs) showed the greatest enrichment. In terms of hormonal regulation, most *GmGH5* promoters contained three types of hormone-responsive elements: ABRE, ERE, and the CGTCA-motif. In addition, all *GmGH5* genes possessed the growth and development-associated element Box4 (ATTAAT). The observed distribution of cis-elements suggests that *GmGH5* genes may be involved in complex biological processes by integrating stress signals, hormonal regulation, and developmental programs.

### 2.6. Spatiotemporal Expression Patterns of GmGH5 Genes Across Different Developmental Stages in the Soybean

**Figure 5:**
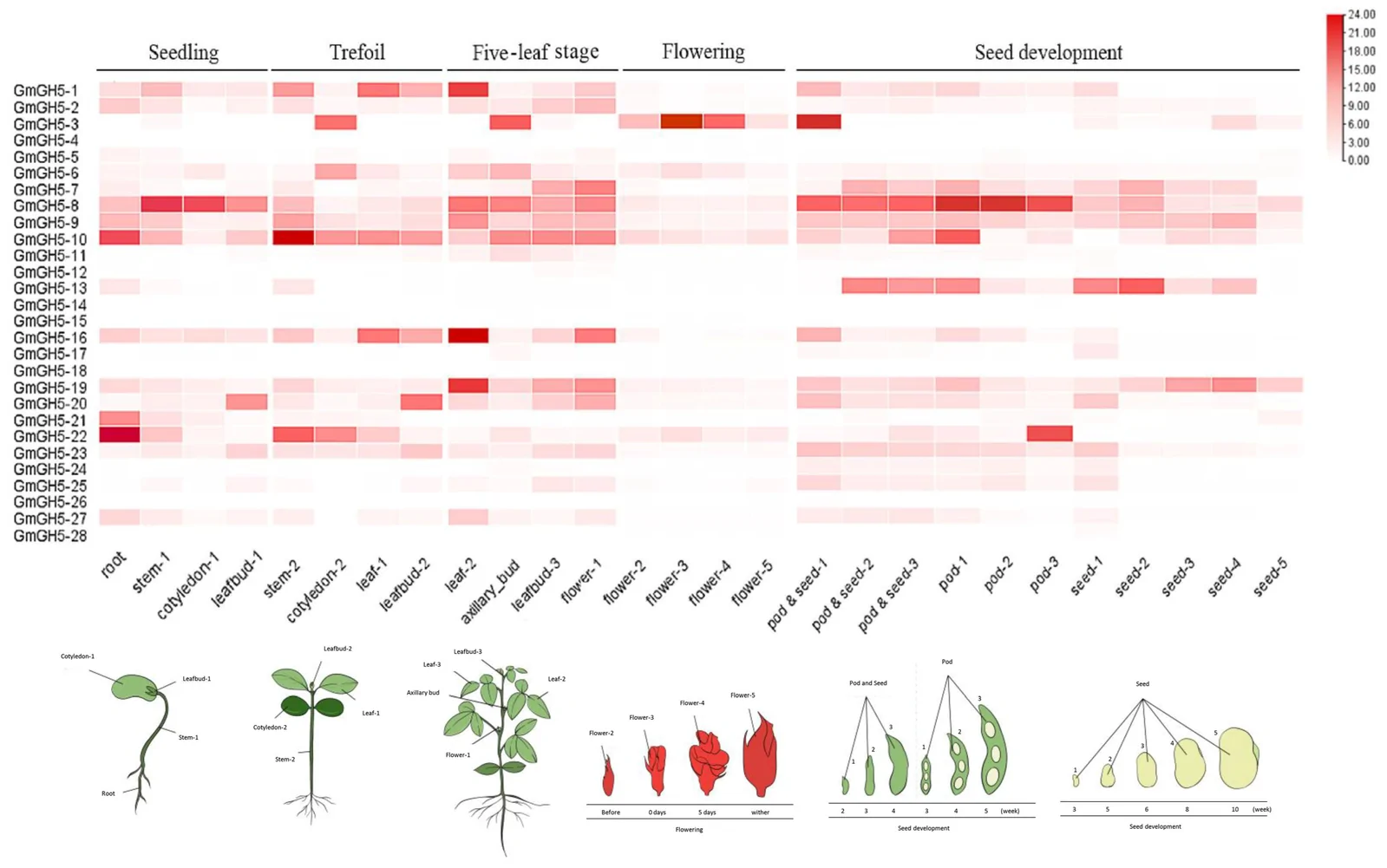
Expression patterns of GmGH5 genes across different organs and developmental stages. The heatmap displays transcript levels (in TPM) obtained from public RNA-seq datasets and was visualized using TBtools. The analysis covers vegetative and reproductive organs (root, stem, leaf, flower, and pod) collected from seedling to maturity. RNA-seq data used for this heatmap were obtained from the soybean cultivar Williams 82.

To elucidate the potential functions of the *GmGH5* gene family during soybean development, we analyzed the expression profiles of 28 members across 27 tissues/developmental stages using the SoyOmics database (Figure 5). The heatmap revealed highly tissue-specific spatiotemporal expression patterns among the family members. At the seedling stage, expression exhibited distinct organ specificity: *GmGH5-22* was preferentially enriched in roots, exhibiting the highest expression level among all tissues in this stage, whereas *GmGH5-8* showed peak expression in stems and cotyledons. Upon entering the trefoil stage, *GmGH5-10* was significantly upregulated in stems. At the five-leaf stage (vegetative growth), *GmGH5-1*, *GmGH5-16*, and *GmGH5-19* were predominantly enriched in the leaf. During the reproductive stage, the expression profiles underwent significant reprogramming. At the flowering stage, *GmGH5-3* was markedly induced. During seed development, expression patterns further diverged: the specific enrichment of *GmGH5-3* shifted from flowers to pods and early seeds (pod & seed-1); *GmGH5-8* exhibited sustained high expression in pods (pod-1 to -3) and early seeds (seed-1 to -3); and *GmGH5-22* showed transient high expression in pods (pod-3). Among these, *GmGH5-22* displayed tissue-specific enrichment in roots at the seedling stage and in pods (pod-3) during late reproduction. This pattern suggests its involvement in root abiotic stress responses and reproductive development.

### 2.7. Expression Patterns of GmGH5s Under Abiotic Stresses

**Figure 6:**
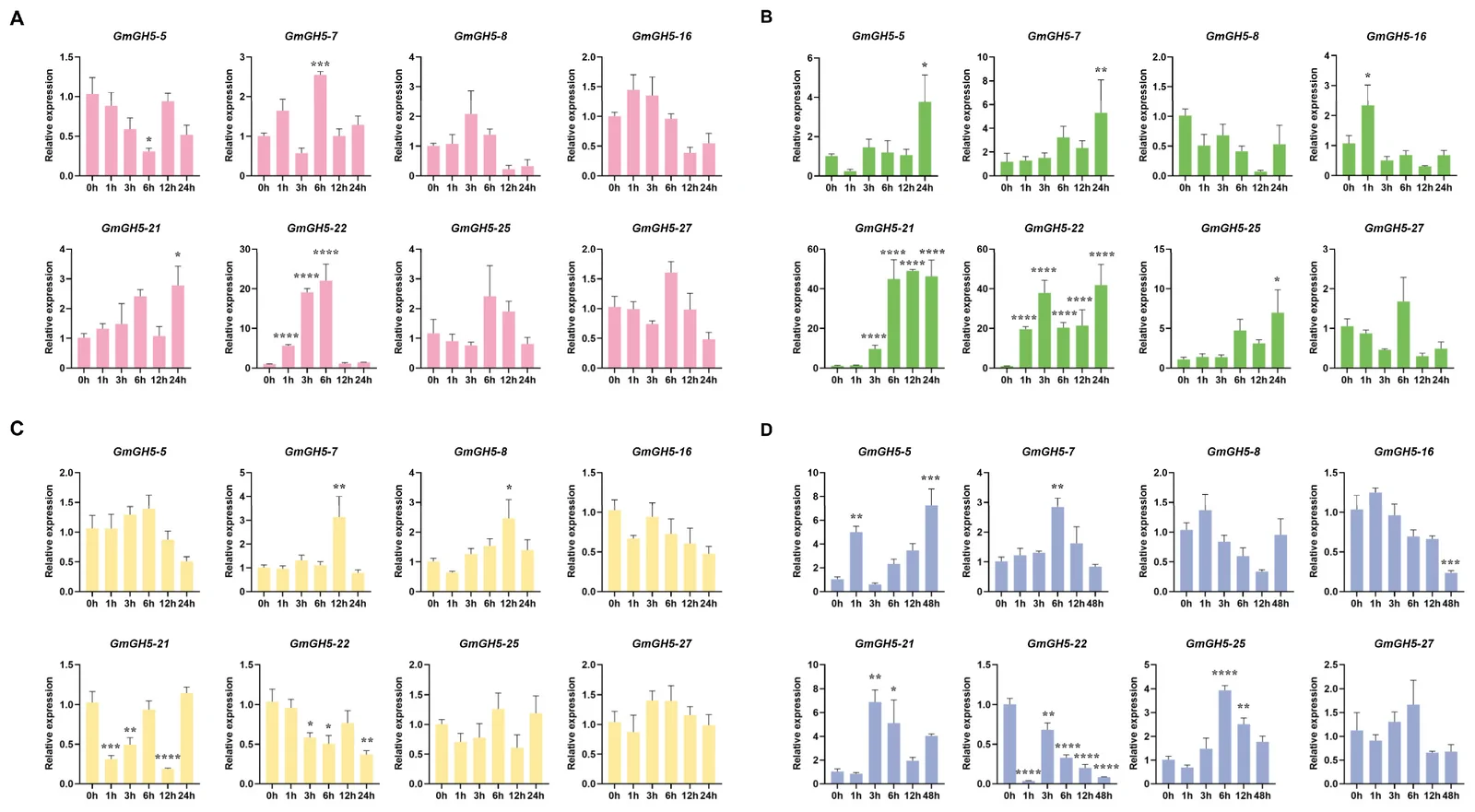
Expression patterns of eight *GmGH5* genes under salt (**A**), alkaline (**B**), cold (**C**), and drought (**D**) stresses. Relative transcript levels at each time point, normalized to *Tubulin* and expressed as fold changes relative to the 0 h control. Error bars represent the standard error of three biological replicates; specifically, error bars at 0 h indicate the biological variation among the independent control replicates, not the variation of the normalized value. Asterisks indicate statistically significant differences compared with the 0 h control (one-way ANOVA, * *p* < 0.05, ** *p* < 0.01, *** *p* < 0.001, **** *p* < 0.0001).

Combining tissue-specific expression data with phylogenetic analysis, eight *GmGH5* genes distributed across three distinct subgroups were chosen for further validation, and their relative transcript levels under salt, alkaline, cold, and drought stresses were determined by RT-qPCR (Figure 6). Under salt stress, *GmGH5-7*, *GmGH5-21*, and *GmGH5-22* were all up-regulated, with *GmGH5-22* showing the most pronounced increases. By contrast, *GmGH5-8*, *GmGH5-16*, *GmGH5-25*, and *GmGH5-27* showed no significant changes (Figure 6A). Under alkaline stress, most *GmGH5* genes in roots were up-regulated except *GmGH5-8* (Figure 6B). *GmGH5-21* and *GmGH5-22* exhibited the most pronounced increases, whereas *GmGH5-27* showed no significant changes. Under cold stress, *GmGH5-7* and *GmGH5-8* were up-regulated, whereas *GmGH5-21* and *GmGH5-22* were down-regulated (Figure 6C). Under drought stress, *GmGH5-5*, *GmGH5-7*, *GmGH5-21*, and *GmGH5-25* were up-regulated, while *GmGH5-22* was significantly down-regulated (Figure 6D). In summary, expression pattern analysis revealed the potential involvement of *GmGH5s*, particularly *GmGH5-22* in salt and alkaline stress responses.

### 2.8. Subcellular Localization of GmGH5-22

**Figure 7:**
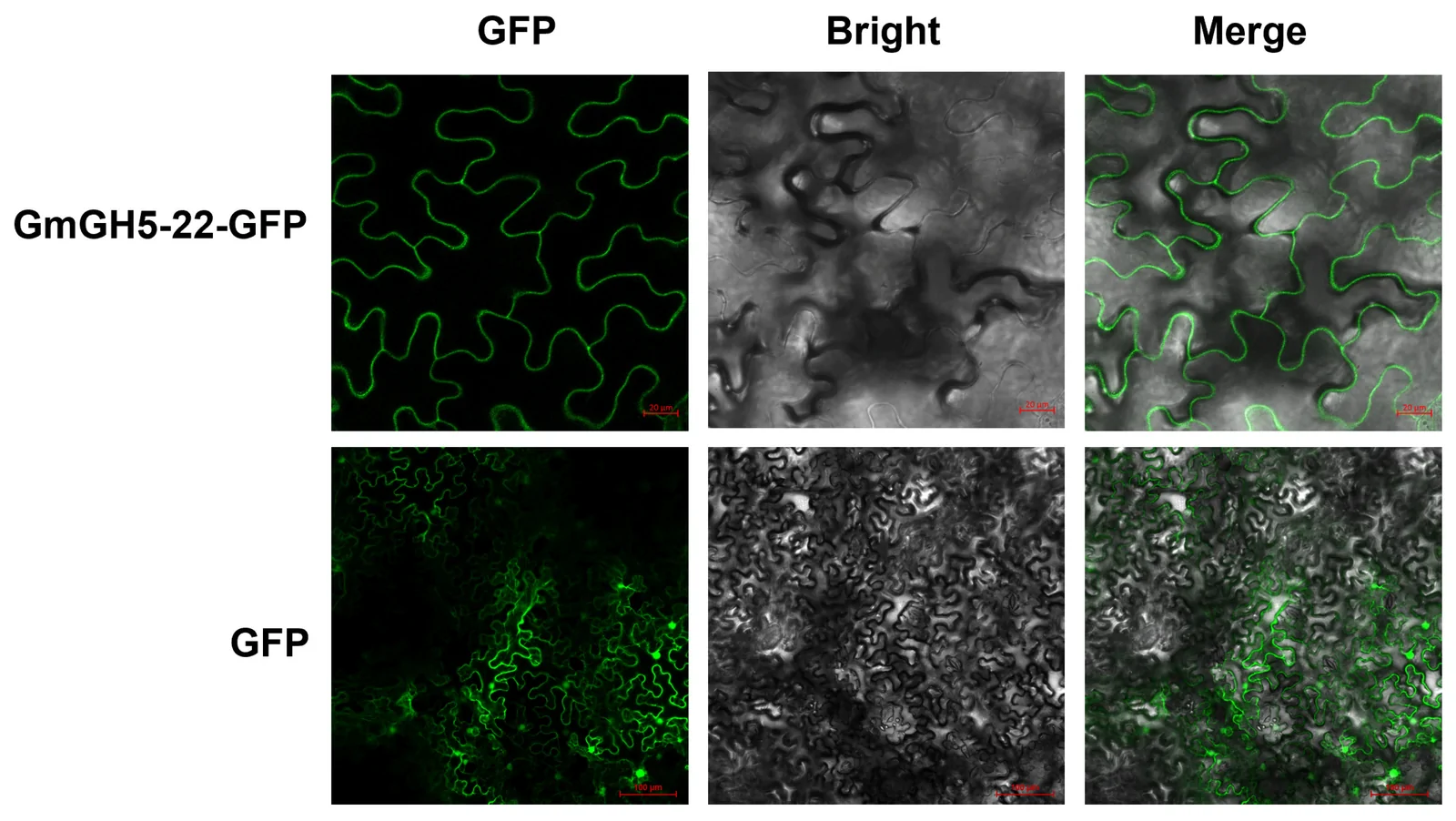
Subcellular localization of GmGH5-22-GFP proteins. The indicated constructs were transiently expressed in tobacco leaves, and fluorescence signals were detected after 3 d.

In silico analyses using SignalP 6.0 for signal peptide prediction, together with DeepLoc, CELLO, and Novopro for subcellular localization, predicted that GmGH5-22 possesses an N-terminal signal peptide and is targeted to the extracellular space (Figure S1), suggesting its potential role as a cell wall-associated protein. To further investigate its subcellular localization, a GmGH5-22-GFP fusion construct was transiently expressed in Nicotiana benthamiana leaves. Confocal microscopy revealed that GmGH5-22-GFP fluorescence was predominantly localized to the cell periphery (Figure 7). Taken together, these complementary results indicate that GmGH5-22 is highly likely a cell wall/extracellular protein.

### 2.9. GmGH5-22 Positively Regulates Salt Stress Tolerance

**Figure 8:**
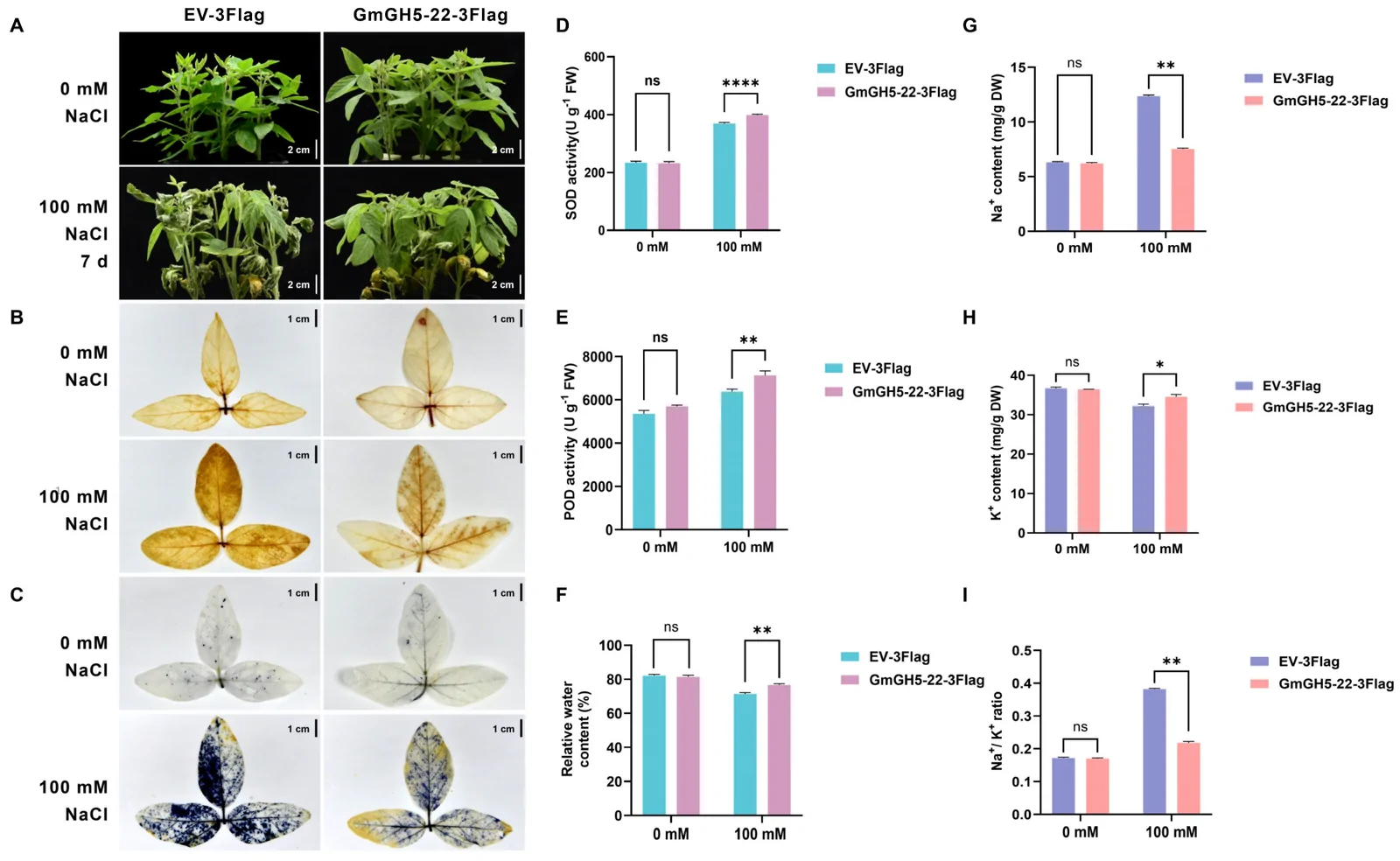
Overexpression of *GmGH5-22* enhances salt tolerance in soybean hairy root composite plants. (**A**) Phenotypes of empty vector (EV-3Flag) and GmGH5-22-overexpressing (GmGH5-22-3Flag) hairy root composite plants under control (0 mM) and 100 mM NaCl treatment for 7 days. (**B**,**C**) Histochemical detection of superoxide (NBT staining, (**B**)) and H_2_O_2_ (DAB staining, (**C**)) in leaves of EV-3Flag and GmGH5-22-3Flag composite plants after 4 days of 100 mM NaCl treatment. (**D**,**E**) SOD activity (**D**) and POD activity (**E**) in the shoots of EV-3Flag and GmGH5-22-3Flag composite plants under control and 100 mM NaCl treatment. At least ten leaves were detached from individual seedlings for each staining assay. (**F**) Relative water content (RWC) in shoots of EV-3Flag and GmGH5-22-3Flag composite plants under control and 100 mM NaCl treatment. (**G**–**I**) Ion contents in hairy roots of soybean composite plants after 24 h salt treatment (100 mM NaCl). The ion content of normal and NaCl-treated root samples was analyzed. The values of Na^+^ (**G**) and K^+^ (**H**) content together with the ratio of Na^+^/K^+^ (**I**) are presented. DW, dry weight. Data are presented as means ± standard error (SE) from at least three independent biological replicates, each with three technical replicates. Statistical significance was determined by Student’s *t*-test. Asterisks indicate significant differences versus the corresponding control (* *p* < 0.05, ** *p* < 0.01, **** *p* < 0.0001).

To elucidate the role of *GmGH5-22* in salt stress responses, we generated soybean hairy root composite plants transformed with either GmGH5-22-3Flag or the empty vector control (EV-3Flag) and compared their phenotypes under salt stress. The GmGH5-22-3Flag composite plants were confirmed to be transgenic positive by PCR and RT-qPCR (Figure S2). As shown in Figure 8A, following 7 days of 100 mM NaCl treatment, GmGH5-22-3Flag composite plants exhibited significantly less severe leaf wilting and chlorosis than the EV-3Flag. DAB and NBT staining revealed lower accumulation of H_2_O_2_ and O_2_^−^ in leaves of GmGH5-22-3Flag composite plants compared with EV-3Flag (Figure 8B,C). Moreover, GmGH5-22-3Flag composite plants exhibited significantly higher SOD and POD activities than EV-3Flag (Figure 8D,E). In addition, GmGH5-22-3Flag plants showed a significant increase in shoot RWC relative to EV-3Flag under salt stress (Figure 8F). Furthermore, ion content analysis in roots revealed that GmGH5-22-3Flag hairy roots accumulated significantly less Na^+^ and maintained higher K^+^ levels than EV-3Flag under salt stress, resulting in a substantially lower Na^+^/K^+^ ratio (Figure 8G–I). Consistent with the shoot phenotype, GmGH5-22-3Flag composite plants exhibited better root morphology under salt stress (Figure S3A) and significantly higher SOD and POD activities in roots than the EV-3Flag (Figure S3B,C). Our results showed that *GmGH5-22* overexpression enhanced salt tolerance.

## 3. Discussion

The GH5 gene family, characterized by the conserved GH5 domain, has been implicated in various biological processes, including plant growth, development, and abiotic stress responses [23,24]. In this study, a genome-wide analysis identified 28 GH5 genes in the soybean. We further characterized *GmGH5-22* as a positive regulator of salt tolerance, as its overexpression significantly enhanced plant resistance to salt stress. This study not only elucidates the evolutionary landscape of the *GmGH5* family but also provides a promising candidate gene for improving crop salinity tolerance.

To provide evolutionary context for the GH5 family in the soybean, the GH5 family in the soybean (this study) is compared with those in rice and Arabidopsis based on previous reports. Soybean contains 28 GH5 genes, versus 17 in rice [24] and 8 in Arabidopsis, suggesting a striking lineage-specific expansion in the paleopolyploid soybean genome [28]. The four-group topology (Groups I–IV) is fully retained in rice, but Groups III and IV are absent in Arabidopsis, and Group IV is also lost in the soybean, suggesting that the four-group structure predates monocot–dicot divergence, followed by independent dicot losses. Group I contains the majority of soybean GH5 members (16/28, 57.1%) and has expanded prominently in the Glycine lineage. In this study, soybean GH5 genes displayed diverse tissue-specific expression patterns, with several members (e.g., *GmGH5-22*) showing notable root enrichment. In rice, individual GH5 members have been reported to be expressed in shoots, leaf sheaths, anthers, and seeds [23,24,29]. In Arabidopsis, individual GH5 members have been shown to be expressed in roots, stems, leaves, seeds, and inflorescences [30,31,32], although systematic expression profiling across the entire family is lacking. We further examined the potential involvement of GH5 members in stress responses at the transcriptional level. In this study, several soybean GH5 members exhibited transcriptional changes under salt, alkaline, cold, and drought stresses. In rice, both a stress-inducible GH5 β-glucosidase (*GH5BG*) and GH5_11 subfamily genes have been shown to be transcriptionally regulated by multiple abiotic stresses [23,33]. In Arabidopsis, GH5_11 subfamily members have been reported to be transcriptionally regulated by multiple abiotic stresses [33], while heterologous expression of the GH5 mannanase gene MirMAN from *Mirabilis jalapa* in Arabidopsis enhanced root development and salt tolerance, suggesting a functional role for GH5 enzymes in abiotic stress adaptation [22]. Collectively, these findings set the stage for a detailed analysis of the expression, evolution, and potential stress-related functions of the *GmGH5* members.

The 28 GmGH5 proteins exhibit striking diversity in protein length, domain architecture, and physicochemical properties, suggesting potential functional divergence. This diversity is further reflected in their protein targeting features. Specifically, 23 members possessed N-terminal signal peptides, whereas the remaining five, which lacked signal peptides, all contained transmembrane domains (9–35 aa), suggesting that most GmGH5 proteins are targeted to the secretory pathway, either as secreted proteins or as membrane-anchored forms, a hallmark of the plant secretome [34]. Two members, GmGH5-12 and GmGH5-17, additionally harbored an RICIN (R-type lectin) domain, indicating a modular architecture potentially involved in carbohydrate recognition. Similar two-domain proteins composed of a glycoside hydrolase and a ricinB lectin domain have been identified as a novel class of plant proteins with synergistic catalytic and binding activities [35]. Consistent with this notion, subcellular localization predictions revealed that GmGH5 members are predominantly distributed in the cytoplasm (16 members) and the cell wall (10 members), with only a few members predicted to localize to the nucleus (2 members). Notably, several members showed dual or even triple localization predictions, involving the chloroplast, vacuole, plasma membrane, and Golgi apparatus, indicating that a subset of GmGH5 proteins may function in multiple compartments. This heterogeneous compartmental distribution suggests functional divergence among GmGH5 paralogs, consistent with the view that plant glycosyl hydrolases have undergone extensive subcellular retargeting during evolution [36].

The subgroup classification was further corroborated by analyses of conserved motifs and gene structures. MEME analysis predicted 15 conserved motifs (Motif 1–15), which were largely shared among members of the same clade but varied markedly between subclades. Likewise, the GmGH5 genes contained 2 to 7 introns, with intron number and length exhibiting subfamily-dependent variation: genes within the same subfamily shared similar intron structures, whereas those from different subfamilies diverged substantially. Taken together, this clade-specific conservation at both the motif and exon–intron levels provides independent support for the phylogenetic grouping and implies that members of the same subclade are likely to perform similar biological functions. The 28 *GmGH5* genes were non-randomly distributed across 13 chromosomes, with tandem arrays observed at several loci (e.g., *GmGH5-3/GmGH5-4* and *GmGH5-5/GmGH5-6/GmGH5-7*). However, synteny analysis identified 24 segmental duplication gene pairs, substantially outnumbering tandem events, indicating that inter-chromosomal segmental duplications served as the primary driver of *GmGH5* family expansion, a pattern consistent with findings in numerous soybean gene families [37,38]. The presence of interconnected gene modules across multiple chromosomes, such as the cluster linking *GmGH5-1* (Chr01), *GmGH5-8* (Chr06), *GmGH5-16* (Chr11), *GmGH5-24* (Chr14), and *GmGH5-25* (Chr17), further supports the retention of duplicates following whole-genome duplication events, which is consistent with the well-documented WGD history of soybeans that has experienced at least two rounds of polyploidization [39]. Robust duplication pairs on homeologous chromosomes, including *GmGH5-1/GmGH5-16* and *GmGH5-2/GmGH5-27*, reinforce the scenario that large-scale genomic duplications have substantially shaped the current *GmGH5* gene repertoire.

The promoters of GmGH5 genes harbour diverse cis-regulatory elements associated with stress responsiveness (e.g., MYB, MYC, AREs), hormone signalling (ABRE, ERE, CGTCA-motif), and development (Box4). This arrangement suggests that GmGH5 genes may function as regulatory hubs integrating environmental cues with endogenous developmental programmes, a feature commonly observed in gene families involved in cell wall metabolism [8]. The *GmGH5* members showed highly tissue-specific expression patterns. *GmGH5-22* was predominantly expressed in roots, whereas *GmGH5-1/19/16* were enriched in leaves, and *GmGH5-3/8* in pods and seeds. This expression divergence suggests the possibility that segmental duplications, followed by subfunctionalization or neofunctionalization, may have partitioned ancestral functions among duplicated copies [40,41], although direct evidence for such functional partitioning awaits enzymatic and transgenic assays. Stress-dependent expression profiling of eight selected members was consistent with this functional diversification. Salt and alkaline stresses induced an overlapping set of genes including *GmGH5-22*, suggesting their involvement in ionic stress responses. Cold and drought, however, triggered opposing patterns, with *GmGH5-22* being repressed under these conditions. This stress-specific regulatory logic may be partly explained by the differential distribution of ABRE, DRE [42,43] and MYB elements in individual promoters, although these predictions require experimental validation to confirm their actual contribution to stress-responsive regulation. Collectively, the promoter architecture and expression divergence are consistent with the functional diversification of the GmGH5 family, wherein distinct paralogs appear to have acquired specialized roles in both development and stress adaptation. Among the stress-inducible members, *GmGH5-22* was prioritized for functional characterization based on its sustained upregulation under salt and alkaline stresses and its root-preferential expression.

The salt tolerance of *GmGH5-22*-overexpressing hairy roots was correlated with multiple physiological improvements. These observations establish *GmGH5-22* as a functional positive regulator of salt tolerance in this transient system. Regarding the underlying mechanism, as a putative glycoside hydrolase, *GmGH5-22* may participate in cell wall remodelling. One possible model is that this process releases oligosaccharides into the apoplast, where they are recognized as DAMPs by membrane-localized receptors, which in turn elicit downstream defence signalling, including the upregulation of antioxidant systems [44,45]. This hypothesis finds indirect support from earlier studies: the salt-inducible rice GH5 β-glucosidase *GH5BG* is secreted to the apoplast and exhibits broad substrate specificity toward various β-linked oligosaccharides [23]; similarly, heterologous expression of the GH5 β-mannanase MirMAN in Arabidopsis enhanced salt tolerance with elevated SOD, POD, and CAT activities and reduced ROS accumulation [22]. Consistent with these reports, our observations of elevated SOD and POD activities in *GmGH5-22*-overexpressing hairy roots suggest that a conserved role for certain GH5 enzymes in reinforcing antioxidant capacity may operate across species. These findings suggest that *GmGH5-22* may play a role in abiotic stress tolerance and that cell wall-derived signalling may be involved in salt stress responses in crops. Nevertheless, because the present study was conducted using a transient hairy root transformation system, the observed phenotypes await confirmation in stable transgenic lines, and the molecular mechanism, particularly the proposed DAMP-mediated signalling, remains speculative and requires further investigation.

## 4. Materials and Methods

### 4.1. GmGH5 Identification and Physicochemical Properties Analysis

The Hidden Markov Model (HMM) profile of the GH5 family (Pfam accession: PF00150) was retrieved from the Pfam database (http://pfam.xfam.org/) (accessed on 2 February 2025) [46]. Using HMMER3.0 [47], the HMM profile was employed to search against the soybean proteome to identify candidate GH5 genes. Candidate sequences were further validated by analyzing the presence of GH5-specific catalytic domains using SMART [48] and CDD [49]. Signal peptides were predicted using SignalP-5.0 (http://www.cbs.dtu.dk/services/SignalP/) (accessed on 4 March 2025). Transmembrane domains were predicted using TMHMM-2.0 (http://www.cbs.dtu.dk/services/TMHMM/) (accessed on 19 March 2025). Biochemical characteristics of GmGH5 proteins, including protein length, molecular weight (MW) and theoretical isoelectric point (pI), were computed using TBtools [50] with default settings. Subcellular localization of soybean GH5 family members was predicted using Plant-mPloc [51] (http://www.csbio.sjtu.edu.cn/bioinf/) (accessed on 22 March 2025). To further characterize GmGH5-22, its signal peptide and subcellular localization were predicted using SignalP 6.0 (https://services.healthtech.dtu.dk/services/SignalP-6.0/) (accessed on 4 April 2025), DeepLoc 2.0 (https://services.healthtech.dtu.dk/services/DeepLoc-2.0/) (accessed on 20 April 2026), CELLO (http://cello.life.nctu.edu.tw/) (accessed on 2 June 2026), and the Novopro protein localization tool (https://www.novopro.cn/tools/protein-localization.html) (accessed on 2 June 2026).

### 4.2. Chromosomal Localization and Phylogenetic Analysis

Chromosomal localization was performed using the GTF/GFF feature of TBtools software, based on the genome annotation file of the soybean. Gene density along the chromosome was calculated using the Gene Density Profile tool (TBtools-II, Version 128, Windows-x64).

The protein sequences of GH5s from *Glycine max* (Wm82.a4.v1), *Oryza sativa* (MSU v7.0), and *Arabidopsis thaliana* (TAIR10) were retrieved from the Phytozome database (https://data.jgi.doe.gov/refine-download/phytozome) (accessed on 4 January 2025). The neighbor-joining (NJ) algorithm was adopted to construct an unrooted phylogenetic tree. The bootstrap test was performed with 1000 replicates. All other parameters were kept at default settings.

### 4.3. Collinearity Analysis of GmGH5s

Collinearity analysis of soybean GH5 genes was performed using Matrix MCScanX (TBtools-II, Version 128, Windows-x64), and the results were visualized using TBtools-II [50].

### 4.4. Analysis of Cis-Acting Elements

The 2000 bp genomic sequences upstream of the transcription start site (TSS) of each gene were retrieved from the soybean genome using TBtools software [50]. Cis-acting regulatory elements located in these promoter regions were predicted and characterized using the PlantCARE online tool (https://bioinformatics.psb.ugent.be/webtools/plantcare/html/) (accessed on 21 June 2025) [52]. The resulting annotations were visualized using TBtools-II [50].

### 4.5. Analysis of Tissue-Specific Gene Expression

Raw expression data of *GmGH5s* in different tissues were downloaded from the SoyOmics online database (accession number: CRA008947; https://ngdc.cncb.ac.cn/soyomics/index) (accessed on 29 June 2025). This dataset comprises multiple soybean accessions, including the reference cultivars ZH13 and Wm82, as well as accessions classified as Cultivar (SoyC01–SoyC14), Landrace (SoyL01–SoyL09), and Wild (SoyW01–SoyW03 and W05) in the database. Gene expression heatmaps were drawn using TBtools-II [50].

### 4.6. Plant Materials and Stress Treatments

Seeds of soybean cultivar DN50 were surface-sterilized with 10% NaClO for 3–5 min, rinsed with distilled water, and germinated for 1–3 days. The germinated seeds were then transplanted into vermiculite and cultured until cotyledon expansion. Subsequently, soybean seedlings were transferred to 1/4 Hoagland nutrient solution and grown hydroponically until the V2 stage. The V2 growth stage was defined as the stage at which the second trifoliate leaf on the third node was fully expanded [53]. Under our growth conditions, plants reached this stage at 16 days after sowing. Uniform V2-stage soybean seedlings were exposed to salt (200 mM NaCl), alkaline (50 mM NaHCO_3_), drought (20% PEG 6000), or cold (4 °C). Stress initiations were staggered so that roots corresponding to the 0, 1, 3, 6, 12, and 24 h post-treatment time points were harvested simultaneously (0 h = untreated control). All samples were snap-frozen in liquid nitrogen and stored at −80 °C. Three biological replicates and three technical replicates were included for each time point.

### 4.7. RNA Extraction and RT-qPCR

Total RNA was isolated via the TRIzol method [54], and the concentration and integrity were evaluated with a NanoDrop spectrophotometer (GE Healthcare, Little Chalfont, UK). RNA integrity was detected by 1.2% agarose gel electrophoresis. cDNA was synthesized using 5× HiScript III RT SuperMix (Vazyme Biotech Co., Ltd., Nanjing, China). RT-qPCR was performed using gene-specific primers with *Tubulin* as the internal reference gene (listed in Table S1). RT-qPCR was performed using SYBR Green qPCR Mix (Thermo Fisher Scientific, Waltham, MA, USA), with 30 cycles set. The amplification efficiency and specificity for each primer set were confirmed by amplification curve and melting curve analysis, respectively (see representative image in Figure S4). Gene expression was measured by the 2^−∆∆Ct^ method. Three independent biological replicates were analyzed, each with three technical replicates, and the results were expressed as the mean of three biological replicates. Statistical significance was analyzed using GraphPad Prism 10 software, and differences were considered significant at *p* < 0.05.

### 4.8. Subcellular Localization Analysis of GmGH5-22

The full-length coding region of GmGH5-22 without the stop codon was cloned and inserted into the pGreen II-62-SK-GFP vector to express the GmGH5-22-GFP fusion proteins. The empty GFP was used as a control. The indicated constructs were introduced into the Agrobacterium tumefaciens GV3101 + p19, which was then infiltrated into *Nicotiana benthamiana* leaves for 3 d. Fluorescence was collected using the confocal laser-scanning microscope (Leica SP8, Wetzlar, Germany), with an excitation wavelength of 488 nm and an emission wavelength of 514 nm.

### 4.9. Plasmid Construction and Soybean Hairy Root Transformation

The coding sequence of *GmGH5-22* was amplified from DN50 soybean cDNA using KOD-Plus-Neo (TOYOBO Co., Ltd., Osaka, Japan) with the primers listed in Table S1. The amplicon was cloned into the plant expression vector pCAMBIA3301-3Flag, and the resulting construct (pCAMBIA3301-GmGH5-22-3Flag) was verified by sequencing at Sangon Biotech (Shanghai) Co., Ltd, Shanghai, China. The verified recombinant plasmid and the empty vector control were introduced into *Agrobacterium rhizogenes* strain K599 via freeze–thaw transformation. DN50 soybean seeds were grown in vermiculite for five days, after which the main roots were excised. For Agrobacterium-mediated transformation, bacterial cells were harvested and resuspended in infection buffer (1 mmol·L^−1^ MgCl_2_, 0.5 mmol·L^−1^ MES, and 100 μmol·L^−1^ acetosyringone) to an OD_600_ of 1.2, and the suspension was injected into the hypocotyl near the cotyledonary node. After infection, the seedlings were transplanted into vermiculite and cultured in a growth chamber with 70% relative humidity under a 16 h light/8 h dark photoperiod at 25 °C. Three weeks post-transformation, positive hairy roots were identified by PCR using *GmGH5-22*-F and NOS-R (Table S1) and then used for RT-qPCR to quantify target gene expression as described above.

### 4.10. Salt Stress Tolerance Assays of GmGH5-22-Overexpressing Soybean Hairy Roots

To evaluate the role of *GmGH5-22* in salt stress tolerance, transgenic soybean hairy root composite plants overexpressing *GmGH5-22* and empty-vector control plants were generated as described above. For stress treatment, plants were grown in 1/4 Hoagland solution for 1 d and then shifted to 1/4 Hoagland solution containing 100 mM NaCl. Each hydroponic box held 12 seedlings, with six boxes per construct and treatment. The new compound leaves were detached from hairy root composite soybean plants on the fourth day of treatment and subjected to nitro blue tetrazolium (NBT) and 3,3′-diaminobenzidine (DAB) staining according to the published protocol [55].

Superoxide dismutase (SOD) activity was assayed by the NBT photoreduction inhibition method [56]. Leaf samples (50 mg) were homogenized in 0.05 M PBS (pH 7.8) to obtain the enzyme extract. The extract was mixed with reaction solution containing 13 mM methionine, 75 μM NBT, 10 μM EDTA-Na_2_, and 2 μM riboflavin. After incubation at 25 °C for 25 min under light, absorbance was read at 560 nm. One unit of SOD is defined as the amount of enzyme causing 50% inhibition of NBT reduction.

Peroxidase (POD) activity was determined by the 2-methoxyphenol method [57]. Leaf samples (50 mg) were homogenized in 0.05 M PBS (pH 7.8) to prepare the enzyme extract. The extract was mixed with 0.4% H_2_O_2_ and 0.01 M 2-methoxyphenol. POD catalyzes the oxidation to 4-o-methylphenol, monitored at 470 nm. One unit of POD is defined as a 0.01 increase in absorbance per minute.

For relative water content (RWC) measurement, shoot fresh weight (FW) was recorded immediately after excision. Samples were then oven-dried at 105 °C for 30 min, followed by drying at 80 °C until constant weight to obtain dry weight (DW). RWC was calculated as RWC (%) = [(FW − DW)/FW] × 100.

For Na^+^ and K^+^ content measurement, the soybean hairy roots of GmGH5-22-3Flag and EV-3Flag composite plants were harvested after 24 h of 100 mM NaCl treatment. Root samples were washed three times with deionized water, subjected to 100 °C for 20 min to deactivate enzymes, dried at 65 °C for 24 h, and ground to a fine powder. The Na^+^ and K^+^ concentrations were determined by Nanjing Weibairui Testing Technology Co., Ltd. using a flame photometer. The Na^+^/K^+^ ratio was calculated accordingly. Three independent biological replicates were performed for each genotype.

### 4.11. Data Analysis

All statistical analyses were performed using GraphPad Prism 10 Software. Data are presented as means ± SE from at least three independent biological replicates. For experiments involving a single independent variable, one way analysis of variance (ANOVA) was applied. For experiments involving two independent variables (e.g., construct and treatment), comparisons between two groups (GmGH5-22-3Flag vs. EV-3Flag) were performed using Student’s *t*-test under each condition. In both cases, a *p*-value < 0.05 was considered statistically significant, with significance levels denoted as *p* < 0.05 (*), *p* < 0.01 (**), *p* < 0.001 (***), and *p* < 0.0001 (****), while *p* ≥ 0.05 was regarded as not significant (ns).

## 5. Conclusions

This study identified 28 GmGH5 family genes in the soybean through genome-wide analysis, and phylogenetic analysis grouped them into three subfamilies. Analysis of the exon–intron organization, conserved domains and motifs, gene duplication events and protein sequence identity suggested conservation of the GmGH5 family within the soybean. Expression analysis revealed the possible involvement of GmGH5s, especially *GmGH5-22*, in soybean responses to salt stresses. *GmGH5-22* was highly expressed in roots, and was highly likely to be localized to the cell wall/extracellular space. Overexpression of *GmGH5-22* in soybean hairy roots enhanced tolerance to salt stresses. Taken together, these findings establish a foundation for elucidating the roles and regulatory mechanisms of *GmGH5s*, particularly *GmGH5-22*, in response to salt stresses.

## Figures and Tables

**Table 1:** Predicted physicochemical properties, transmembrane regions, and subcellular localization of GmGH5 proteins.

Name	Accession ID	Protein Length/aa	MW/kDa	pI	SP/aa	TMD/aa	Subcellular Localization
*GmGH5-1*	*Glyma.01G171900*	431	49.28973	9.44	1–20	-	Cytoplasm
*GmGH5-2*	*Glyma.03G002100*	463	52.63043	5.72	1–27	-	Chloroplast/Cytoplasm
*GmGH5-3*	*Glyma.03G226000*	410	46.09428	7.77	1–28	-	Cytoplasm
*GmGH5-4*	*Glyma.03G229100*	415	47.54283	6.87	1–23	-	Cytoplasm
*GmGH5-5*	*Glyma.05G173300*	571	63.14807	5.64	1–17	-	Cell wall
*GmGH5-6*	*Glyma.05G173400*	557	61.91702	6.32	-	9~28	Cell wall
*GmGH5-7*	*Glyma.05G180700*	503	57.1192	5.71	1–27	-	Cell wall
*GmGH5-8*	*Glyma.06G030500*	420	47.42636	7.1	1–20	-	Cytoplasm
*GmGH5-9*	*Glyma.06G292400*	436	50.47663	7.11	-	13~35	Cytoplasm
*GmGH5-10*	*Glyma.06G319460*	500	56.23793	5.49	1–16	-	Cell wall
*GmGH5-11*	*Glyma.08G060700*	532	59.79396	6.33	1–22	-	Nucleus
*GmGH5-12*	*Glyma.08G130600*	575	63.11597	5.53	1–32	-	Cell wall
*GmGH5-13*	*Glyma.08G138300*	503	56.74876	5.24	1–23	-	Cell wall/Vacuole
*GmGH5-14*	*Glyma.08G242200*	433	48.35225	6.93	1–23	-	Nucleus
*GmGH5-15*	*Glyma.09G224500*	402	46.05827	5.45	1–25	-	Cytoplasm
*GmGH5-16*	*Glyma.11G071300*	426	48.87332	9.54	1–20	-	Cytoplasm
*GmGH5-17*	*Glyma.11G179700*	545	60.84903	6.71	1–29	-	Cell wall/Nucleus
*GmGH5-18*	*Glyma.12G012500*	408	46.22843	5.65	1–25	-	Cytoplasm
*GmGH5-19*	*Glyma.12G113400*	437	50.33649	7.14	-	13~35	Cytoplasm
*GmGH5-20*	*Glyma.12G204700*	429	49.10667	5.97	-	7~26	Cytoplasm
*GmGH5-21*	*Glyma.13G087400*	509	57.42235	8.15	1–28	-	Plasma membrane/Cell wall/Golgi apparatus
*GmGH5-22*	*Glyma.13G087500*	507	57.01447	7.73	1–27	-	Cell wall
*GmGH5-23*	*Glyma.13G296600*	429	49.10767	5.79	-	7~26	Cytoplasm
*GmGH5-24*	*Glyma.14G072600*	430	48.63588	5.95	1–28	-	Chloroplast/Cytoplasm
*GmGH5-25*	*Glyma.17G252400*	433	48.97812	5.48	1–28	-	Cytoplasm
*GmGH5-26*	*Glyma.18G012900*	534	59.23392	6.41	1–31	-	Plasma membrane/Cell wall
*GmGH5-27*	*Glyma.18G215600*	463	52.49036	5.59	1–23	-	Cytoplasm
*GmGH5-28*	*Glyma.19G226300*	419	47.65612	5.96	1~19	-	Cytoplasm

## Data Availability

The data that support the findings of this study are avail-able from the corresponding authors upon reasonable request due to privacy.

## Supplementary Materials

The following supporting information can be downloaded at https://www.mdpi.com/article/10.3390/plants15172700/s1. Table S1. The primers used in this study. Figure S1. In silico prediction of the subcellular localization and signal peptide of GmGH5-22. (A–C) Subcellular localization of GmGH5-22 predicted by DeepLoc 2.0 (A), CELLO (B), and Novopro (C), respectively. All three independent tools consistently predicted GmGH5-22 to be localized to the extracellular space. (D) Signal peptide prediction using SignalP 6.0. The red line represents the signal peptide probability (n-score), the yellow line represents the hydrophobic region (h-score), and the green vertical dashed line indicates the predicted cleavage site (CS). Figure S2. Verification of *GmGH5-22*-overexpressing soybean hairy roots. (A) PCR identification of *GmGH5-22*-positive transgenic hairy roots. (B) RT-qPCR analysis of the relative expression of *GmGH5-22* in GmGH5-22-3Flag and EV-3Flag soybean hairy roots. Figure S3. Overexpression of *GmGH5-22* enhances salt tolerance in soybean hairy root composite plants (root phenotypes and antioxidant enzyme activities). (A) Root phenotypes of EV-3Flag and GmGH5-22-3Flag hairy root composite plants under control (0 mM) and 100 mM NaCl treatment for 7 days. (B,C) SOD activity (B) and POD activity (C) in the roots of EV-3Flag and GmGH5-22-3Flag composite plants under control and 100 mM NaCl treatment. Figure S4. Representative amplification curve and melting curve for RT-qPCR primers. (A) Amplification curves. (B) Melting curves.
